# Optimization of an appointment scheduling problem for healthcare systems based on the quality of fairness service using whale optimization algorithm and NSGA-II

**DOI:** 10.1038/s41598-021-98851-7

**Published:** 2021-10-06

**Authors:** Ali Ala, Fawaz E. Alsaadi, Mohsen Ahmadi, Seyedali Mirjalili

**Affiliations:** 1 Department of Industrial Engineering & Management, Shanghai Jiao Tong University, Shanghai, 200240 China; 2 Information Technology Department, Faculty of Computing and Information Technology, King Abdulaziz University, Jeddah, Saudi Arabia; 3 Department of Industrial Engineering, Urmia University of Technology, Urmia, Iran; 4 Centre for Artificial Intelligence Research and Optimization, Torrens University Australia, Brisbane, QLD 4006 Australia; 5 Yonsei Frontier Lab, Yonsei University, Seoul, South Korea

**Keywords:** Health care, Engineering, Mathematics and computing

## Abstract

Effective appointment scheduling (EAS) is essential for the quality and patient satisfaction in hospital management. Healthcare schedulers typically refer patients to a suitable period of service before the admission call closes. The appointment date can no longer be adjusted. This research presents the whale optimization algorithm (WOA) based on the Pareto archive and NSGA-II algorithm to solve the appointment scheduling model by considering the simulation approach. Based on these two algorithms, this paper has addressed the multi-criteria method in appointment scheduling. This paper computes WOA and NSGA with various hypotheses to meet the analysis and different factors related to patients in the hospital. In the last part of the model, this paper has analyzed NSGA and WOA with three cases. Fairness policy first come first serve (FCFS) considers the most priority factor to obtain from figure to strategies optimized solution for best satisfaction results. In the proposed NSGA, the FCFS approach and the WOA approach are contrasted. Numerical results indicate that both the FCFS and WOA approaches outperform the strategy optimized by the proposed algorithm.

## Introduction

The fundamental role of healthcare systems is to provide low-cost, timely, and relevant access to health services for all patients. The purposes of such scheduling can be divided into minimizing the cost of services, maximizing patient satisfaction, minimizing waiting time, maximizing Fairness policy, and cost efficiency in healthcare^1^. One of the crucial issues in healthcare is fairness, which is a method of assigning resources to healthcare such that all patients get, on average, an equal share of resources over time when a patient is admitting, that patient uses the complete services this policy always shares between patients (include in regular and emergency one) and doctors. Aside from fairness in appointment scheduling, further encouragement is achieved through a novel gain framework unique to the division and was not reported previously. Another critical issue on fairness is in mending personal scheduling preferences that mean each doctor has his or her scheduling preferences^2^. In making a schedule, these should be met as much as possible. Appointment scheduling is a system that makes access to healthcare facilities manageable and results from efficient and successful healthcare services. In this way, the planning of appointments leads to patient satisfaction. The planning of appointments is used for various sections, initial visits, individual visits, and optional operations. For first clinics, doctors split their time into individual or multiple slots based on their appointments. The rules of access to primary care also depend on patients’ urgency and the quality of services. For example, first emergency cases are scheduled, and patients are regularly visited or followed up. Also, some clinics allow open access systems that designate patients the same day, irrespective of urgency. Besides the advanced access rules, primary care providers should also accept walks daily that can cause other scheduled patients to wait directly^3^. Like primary clinics with equal time slots, time slots have a high variation in specialty clinics. Access regulations help to determine the time for each appointment and possible future urgent appeals^4^. The Whale Optimization Algorithm based on the Pareto Archive and the NSGA-II algorithm is presented to solve the appointment scheduling model using a simulation method. This article explored the multi-criteria technique in appointment scheduling using these two algorithms. This article computes them using multiple assumptions to satisfy the analysis and diverse aspects connected to WOA and NSGA. In the last part of the model, this article evaluated NSGA and WOA with three examples, as well as the fairness policy first come first serve (FCFS), to determine the essential element to consider in order to achieve the most significant satisfaction outcomes from the figure to strategies optimal solution.

The structure of the paper is as following sections. In the “Introduction” section, the background of the problems and motivation of the research are presented. In the “Literature review” section, a brief review of the state-of-the-art research and the advantages and disadvantages of some methods are illustrated. In the “Proposed approach” section, the features of the presented algorithm are illustrated. Moreover, in the “Methods and materials” section, the sequential appointment scheduling process is described. In the proposed structure of the WOA algorithm, a recovery procedure is designed that applies and improves the solutions. Also, in the “Results” section, the proposed method’s finding is depicted in the computational and numerical analysis field. Furthermore, in the “Discussion” section, the overall findings of the presented method are illustrated and compared with other techniques. Finally, in the “Conclusion” section, the summary of the results focusing on the future works is provided.

## Literature review

This section provides a brief review of the state-of-the-art research and the advantages and disadvantages of some methods. Moreover, the results of the papers are explained. Dogru and Melouk^5^ stated that the distinction between in-patients and the hospital’s outpatient is traditionally not so clear. Recently there was a move from hospital to ambulatory care where more patients were treated in space for ambulatory treatments. Takashima et al.^6^ presented that the emergency department is also an entry route. Such patients are usually referred to as emergency patients at the outset of their treatment status. This paper is hospitalized once admitted to a normal unit. This research focuses on outpatient scheduling problems, with limited reference to the most relevant literature on general patient scheduling problems, as these two topics somewhat overlap. On the other hand, Zhang et al.^7^ presented a new numerical programming model for an expanded open shop issue with clinic prices for an Integrated Practice Unit (IPUs). The new model addresses its benefits and presents some essential inequalities to improve linear programming relaxation. The purpose of the issue is to minimize a combination of makeup and overall working time or an IPU to reduce closing time and maximum patient waiting time.

The arrival times for a patient series were called decision indicators by Zhang et al.^8^. The model seeks to reduce the weighted costs generated by a patient’s waiting period, server overtime, and server acceleration due to overcrowding. This paper presented suitable mathematical formulations for this approach as a model for Simulation Optimization (SO) and a Stochastic Integer Programming (SIP) model. Cayirli and Yang^9^ proposed that the influence of these optimization programming factors is calculated using the ideal concept that already changes patients’ appointment times to minimize the adverse effects of these factors to the exclusion of their residual or actual impacts on overall cost efficiency. Sauré et al.^10^ introduced ways to overcome these two challenges; they expose a framework incorporating stochastic service times with the advanced scheduling problem. This way, this paper considers the waiting time before the service day and the free time-workload on the service day of the health care service. It is a standard procedure for a patient to contact the hospital a couple of days in advance to book an appointment the day they decide to see the doctor. The main factor affecting both operating performance and patient satisfaction is timely access to outpatient clinics. Some well-crafted appointment plans would provide adequate care to the patient without increasing Laganga and Lawrence^11^. Thankfully, most healthcare providers face long waiting times. This concept is complex by a no-show, variability of service time, and the patient’s desired appointment. The healthcare staff’s work environment significantly influences the patients’ health. Therefore, strengthening these conditions will contribute to the effectiveness of the treatment process being improved. It is understood that productive workforce efficiency has always been a vital concern of any enterprise and one of the most effective methods of achieving productivity benefits. For this reason, staff and patient scheduling are a field that has become particularly critical in certain high-demand and often restricted staff healthcare facilities. As the healthcare requirement increases, appointment scheduling significantly impairs the capacity utilization of healthcare care and service quality. However, the hospital appointment scheduling is limited by several factors such as lack of spaces, number of beds, outpatient category, and surgical schedule. Considering that these variables complicate appointment scheduling, the conventional fairness strategy for first come-first serve (FCFS) cannot guarantee performance. Also, the two primary objectives of healthcare services, i.e., increasing the capacity utilization of medicinal resources and keeping fairness, usually challenge one another. Thus, an adequate and fair strategy is demanded appointment scheduling of outpatients. As described above, the growing demands for this service require appropriate patient and staff schedules to guarantee the total number of patients benefiting through high labor force use. In addition to increasing the number of patients, balancing the physiotherapist workload, and keeping a daily schedule is often crucial. For them, the case study is performed in the physiotherapy department of the Shanghai United Hospital because of hard-working constraints. Lastly, the selected patients are scheduled throughout a working day to minimize their waiting time. Harper et al.^12^ developed a simulated model for controlling and preparing beds in healthcare centers.

Hutzschenreuter et al.^13^ designed an outpatient flow structure. They implemented an operator method to allow for an optimal mix of patients from different departments. Demeester et al.^14^ built a hybrid Tabu search algorithm that allocates beds in the proper units to outpatient clinics. Both algorithms above are run regularly to do the scheduling job. However, everyday scheduling optimization cannot always assure the algorithm’s improved long-term effect. Zhang et al.^15^ assigned its robustness, and global convergence capability defines it. Thus, the Genetic Algorithm (GA) is fit to solve the problem of appointment scheduling. This paper discusses a new GA for optimizing the selection policy for fairness, separate from all the above strategies in the following paragraphs. In time, the algorithm reviews optimization of a long-term admission plan rather than directly doing the scheduling research. In order to make the equity more reliable than a discrete event simulation model, it is necessary to use discrete event modeling techniques if the mechanism under study can be represented as a series of operations naturally. For instance, agent-based modeling might be the solution if the behavior, rather than attempting to construct a global workflow, is easier to explain than to attempt. Likewise, group dynamics should be extended if you are interested in aggregate values and not in the relationship of individual units. The simulation model measures the value of the normal function, which is contrasted with the mathematical model below. The hospital healthcare system that will be analyzed here has a discrete event structure. As previously mentioned, much software has been released for this purpose, which can be named ANY LOGIC software^16^, one of the most popular professional simulation software. For instance, a new optimization algorithm named WOA has been recently developed by Mirjalili and Lewis^17^ for meta-heuristic algorithms. It is known that whales are the most intelligent aquatic species. Humpback whales’ unique hunting activity is the main inspiration of the WOA algorithm. Humpback whales use a hunting method called the bubble net feeding system. WOA has been widely used in the literature to solve different optimization problems.

Emary et al.^18^ introduced a WOA-based feature choice method. The introduced variants and the original algorithms were benchmarked using unimodal, multimodal, fixed-dimensional, and composite benchmark functions. The test was performed using a series of assessment metrics that show the potential of the proposed models to outperform. Reddy et al.^19^ also implemented an original strategy to solve feeder reconfiguration to minimize significant total loss and optimize energy savings in a spaced distribution network by using the algorithm for whale optimization. Anand and Dinakaran^20^ used WOA to limit the size of the resources of the optimally distributed generator (D.G). Such tools were the small-scale power generation plants that can provide power in the distribution system to households, businesses, or industrial types of equipment. Besides, Nasiri et al.^21^ suggested a WOA-based clustering approach and demonstrated the W.O’s merits. In classifying area too. Although the original version of WOA was successful, some works suggested that its performance could deteriorate when some issues were resolved by Jadhav and Gomathi^22^. The Simulated Annealing (SA) algorithm was applied by Mafarja and Mirjalili^23^ as a local search method to enhance the WOA to choose the optimal subset of features to develop classification accuracy. Ling et al.^24^ used the Lévy Flight for regional optimization to improve the WOA. However, the WOA weight algorithm has been suggested by Dong et al.^25^. The developers hybridize WOA with a pattern analysis technique to overcome maximal power flow problems. Jiang and Deng^26^ showed that the whales attack the prey by circling spirally around them and releasing bubbles in a circular shape. It is established that there are many types of WOA modifications and implementations. It is impossible to compare each new proposed WOA with all other types. There are different benchmark functions, which can be used to test any new modifications. According to the research findings, the Non-dominating Sorted Genetic Algorithm (NSGA-II) and the WOA have been investigated in the healthcare sector. WOA outperforms other standard algorithms in terms of computation time and harmonizing utilization by addressing Pareto optimal solutions. When compared to WOA, its variations and comparison with standard measurements also perform well.

## Proposed approach

**Figure 1:**
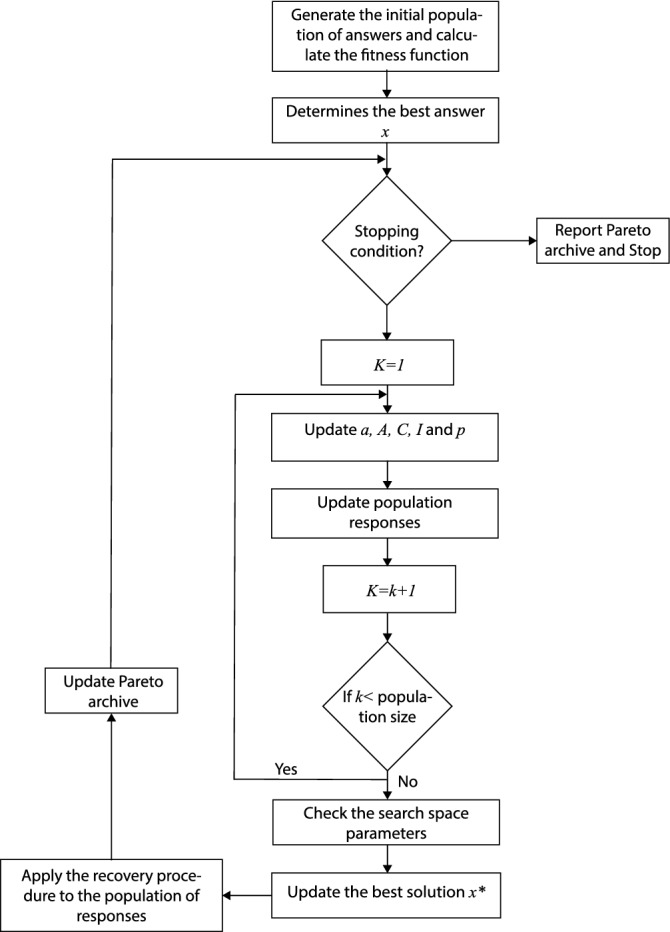
Proposed flowchart for the whale optimization algorithm.

In this section, the features of the presented algorithm are illustrated. We explain the algorithm using AnyLogic professional: V8.7.3^16^, which can simulate and carry out discrete, agent base, and system dynamics simultaneously. Although this software can simulate general numerical spectra of processes, it has many specialized pallets for road transport research issues, Railroads, and even simulations of the transmission lines of liquids, which can be seen in Fig. 1. This paper has introduced a new algorithm for this research based on GA: The whale optimization algorithm (WOA) has the same structure as the traditional WOA for optimizing scheduling techniques, except that a local search operator is introduced to optimize solutions. The WOA algorithm includes three operators modeling humpback whale’s behavior in the beast quest, prey encircling, and bubble-net behavior. In this research, the proposed model is solved with the random WOA essence of the meta-heuristic algorithms, and exactly which algorithm is superior cannot be stated. This paper is also seeking to use relatively new algorithms to solve the model in the current research. By comparing them with the well-known Non-Dominated Sorting Genetic Algorithm (NSGA-II) algorithm, they evaluated their performance scientifically and practically for the problem under investigation.

The WOA starts with a random set of solutions. According to each search factor, the search agents arbitrarily change their location with the best solution obtained in each iteration. Parameter A is lowered from two to zero to provide discovery and exploitation. The location of the quest agents can be updated in two ways. The random search agent is selected if $|A|>1$; otherwise, the best solution is selected. Depending on the p-value, the whale can switch between two spiral and rotational motions. Ultimately, the whale’s algorithm ends after reaching the set satisfaction criterion. The following is the pseudo-code of the algorithm. There are two stages of mining and discovery in the WOA. In the operating phase, the strategy of the bubble-net attack is used to demonstrate the process based on the current optimum search agent. This attack involves two methods, including pride and spiral swimming. Each whale changes its location independently in the discovery process, depending on the randomly selected search agent. As seen in the following tables, the runtime of the algorithms shows that runtime values and runtime comparisons indicate that the multi-objective WOA has a higher resolution time. Because of the proposed structure of the proposed method, this method intelligently searches many points of the answer space in each iteration. This method consumes more computational time than the NSGA-II method. According to the comparison findings, the WOA can supply higher-quality replies than the NSGA-II algorithm. The whale method is better capable of identifying and retrieving the active region than the NSGA-II algorithm. It can provide more excellent dispersion responses than the NSGA-II algorithm.

## Methods and materials

### Description of the sequential appointment scheduling process

**Figure 2:**
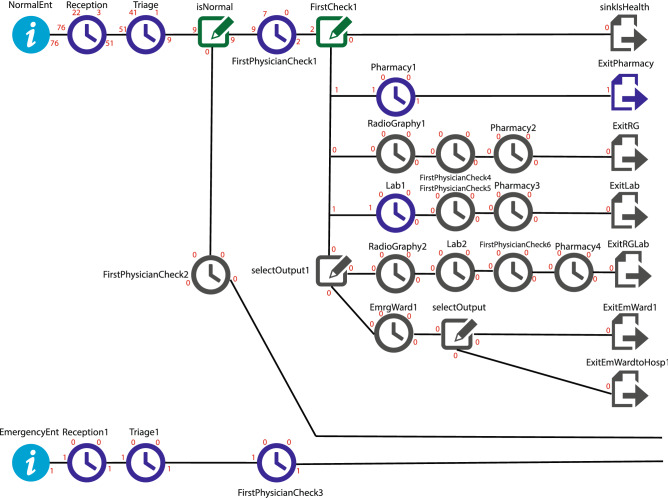
Simulation model of appointment scheduling for two different patient type.

Once the hospital generates outpatient appointment service, the hospital manager is constantly faced with a challenge for how many patients should be accepted when slots are assigned to these patients in the surroundings. A sequence of patients is calling in to request an appointment in the future session. Patients arriving at the hospital are split into two groups: regular patients and emergency hospital admitting patients. Urgent outpatients are often admitted as long as they follow normal patients. The fairness principle is followed. Emergency patients at both stages shall be served without a line upon entering one of the triages, doctors, pharmacy, laboratory, or radiographic units. However, to assume another emergency patient is on the queue list of waiting places. In this case, the newly arriving emergency patient will be put in the emergency service line until the screening of the former emergency patient is complete. The critical aspect to note is that doctors may understand that regular patients should be treated in an emergency during the visit, which has also been considered in the model. The start number for patient servers (referred to in the program as the resource) is used as the default slider number in each section to initiate the method. For further explanation of the model, Ordinary and emergency patients are logged in with the distribution functions stated in the tables in the preceding Fig. 2, marked with *NormalEnt* and *EmergenctEnt*, respectively. The number inserted next to each indicates that several patients of each type were logged in at this particular moment. There were 76 regular patients and one emergency patient. It shows that all 76 patients admitted to the hospital entered the admission ward, and 51 were admitted to the triage ward. The model assumes that the change in fairness policy does not include people being serviced. The upper left number indicates the number mentioned. The high correct number indicates the current busy patients who are checking by doctors. That will be the maximum number of personnel assigned to each department. In the figure above, after a patient is diagnosed in the triage department, the doctor is examined (FirstPhysicianCheck1 station).

**Figure 3:**
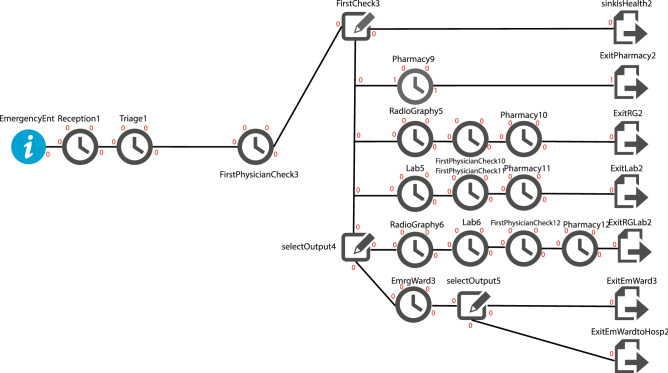
A simulation model for emergency patients with high priority fairness policy.

In this case, several events may occur:

I. The doctor determines that the patient does not need treatment or is healthy and requires no special treatment. The Sink is Health counters the number of these patients.
II. The doctor realizes that the patient will better by getting the drug, so by writing a prescription and following the patient’s medication, whose statistical distribution is listed in the above tables, the patient is an exit from the *ExitPharmacy*, which when taken, is one Logged out of this port.
III. As shown in the figure, the doctor will send the patient to the radiography. After viewing the photograph taken by the physician and taking medicine from the pharmacy, it will be removed from the *ExitRG* port.
IV. Alternatively, the third part of the laboratory will happen instead of the radiography. The patient will eventually exit the *ExitLab* portal.
V. In another case, the patient is referred to because of their specific conditions to the radiographic and laboratory departments. After seeing the results by the doctor, the outpatient then walks to the clinic and leaves the *ExirRGLab* portal after taking the drug.
VI. In the other case, the doctor will prescribe that patient be admitted to the ward or according to the patient’s condition. It should be transported to a specialized hospital where that can take better healthcare services. On the other hand, for an emergency hospital or patients whose doctor determines they have emergency conditions, the route is described for regular patients except for the higher priority (Fairness) considered for this patient segment. None of these people, in the regular client queue, will have the slightest delay, and as soon as they enter each part, they will be dealt with out of the queue, as shown in Fig. 3.

The equity priority for each patient specified in the settings option is set at zero for regular patients and 2 for emergency patients. These two parameters are optional and should be perceived as a higher amount for emergency patients. There are only two types of patients; no matter what name is found will make any difference. Also, emergency patients should be given priority over regular patients. This paper calls the system run with the values of the initial parameters run 0. At this stage, it is observed that this paper has about one efficiency in the acceptance section.

**Figure 4:**
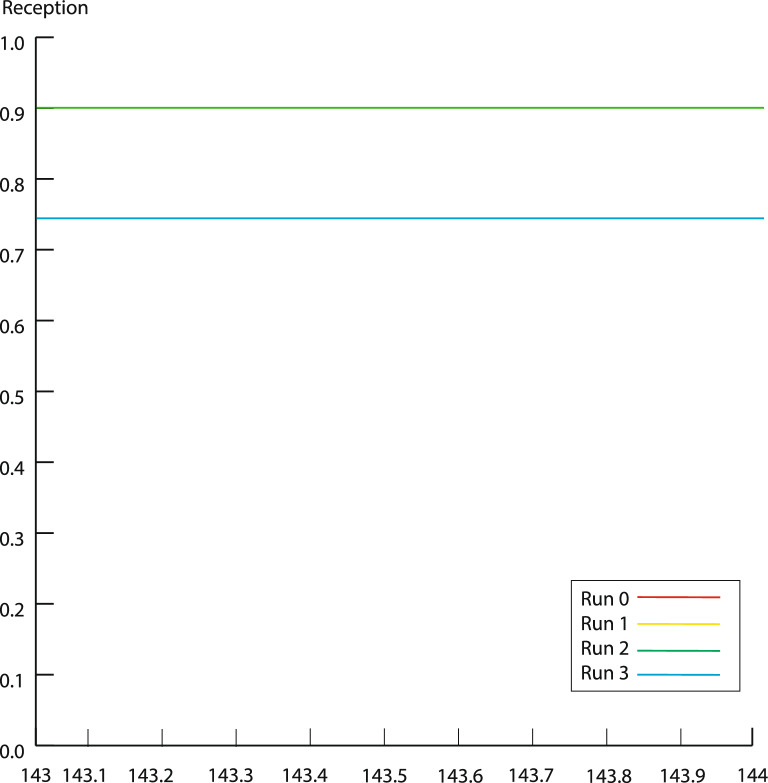
Admission section efficiency in a different run in the admission department.

To determine if an increase in the number of admissions can reduce waiting times and keep performance at a high level, this paper will increase the number of individuals in the admission department in a few steps to maximize the number of people who can keep getting high. In Fig. 4. After raising the number from three to six and hitting the number six (blue), this paper sees a substantial decrease in output. This paper base the number 5 on the research and continue with the simulation model. Moreover, this is what this paper call run 1.

### Problem statement

The difficulty is a fixed number of similar outpatients I scheduled over a D-day planned period. Every other day consists of T-periods, specified in increments of 1 h. This paper suggests a problem of mid-term scheduling, where lineups are produced for up to several weeks. The appointment cannot move over to the next day of preparation. At most, one admits starting per day is permitted in this discontinuous planning problem. The outpatients have some distinctive features, such as minimum waiting period, maximum waiting period, the total time between successive admissions seeing a doctor, waiting time, and limits on overtime per week. According to the general waiting time rules, every shift must be given a lunch break within a specified period. This period is defined by the minimum amount of time on duty before and after the break. Besides, all the changes allocated to one or more physicians may fulfill a specific start time window to account for the patient or general preferences. Due to the number of empty beds and a particular policy like the FCFS, the hospital accepts satisfied patients in the waiting line each day. Provided that doctors and resources are restricted, the surgeries mentioned below are subject to certain constraints.

1. Patients suffering from eye trauma need surgery the day after they arrive. When the condition cannot be met, the patient may be automatically moved to another hospital.
2. Surgical treatment and other operations (except for ocular trauma surgery) should not be performed on the same day.
3. Some cataract patients need only one eye operation, although other patients need both eyes to undergo surgery.

**Figure 5:**
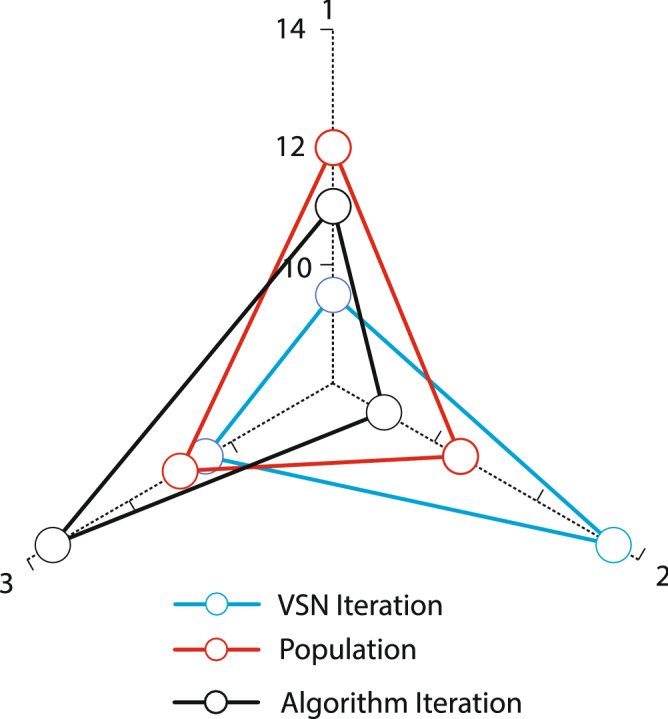
Noise signal in WOA.

First, the latter will have surgery for one eye, and 2 days later, surgery service on the other eye. When a patient is admitted for surgery, the patient must go through 4 stages before leaving the hospital. Figure 5 presents those four levels. Phase 1 is the stage of planning, in which the hospital prepares for the procedure. The patient waits for the surgery during step 2 if the process cannot be scheduled immediately after step 1. It implies that stage 2 is a waste of resources and can be minimized if a proper approach allows the patients to be admitted. Phase 3 is for the surgery, and step 4 is for post-operative resumption. This study suggests that all patients arriving are on time if they appear for their appointment and are presented following a first-in-first rule; the service times are identically and independently distributed exponentially across patients.

As explained above, an outpatient’s situation determines the length of all steps and cannot be optimized. The second phase, however, includes a waste of sources, so it may its time. While wasting a patient’s time and money, step two also allows the bed resource unused to the max. In such healthcare delivery networks, the bed is one of the bottlenecks impacting healthcare services’ productivity levels. Moreover, reducing the average time of stage two improves the serving rate. The policy is fair but potentially ineffective if the department sticks to the fairness policy. The fairness policy will result in a more extended average period of stage two.

On the other hand, if the unit accepts only outpatients with the required minimum stage two times (and this is a greedy strategy), there may be appointment disparity among different patients, which may eventually affect the system’s effectiveness. Furthermore, a strategy other than fairness and greedy is required for an effective and equitable health service. The optimization goal in this work is to find an optimal approach to minimize the waiting time and ensure fair and comfortable treatment of all types of patients. This paper refers to GA. To the question of seeking an efficient and equitable appointment scheduling method rather than directly scheduling the appointment. Therefore, the algorithm is tested only once for a suitable strategy to compare with the simulation of discrete events to determine the policy will be fair and equal to accept. All the scheduling work is done daily following the optimized strategy.

The overall problem was split into three sequential and logical levels. It was pretty tricky and impractical for even small-size test problems to solve the entire problem in a single phase within a reasonable time. As described, arranging and consulting the patient scheduling to a clinic is the issue under discussion. It is assumed that the hospital has the *M* department. Each section has a different number of operators to process different operations. Also, each section can only perform some services. This system has n patients, some referring to emergency patients and some as regular patients. Each patient has several operations that must be completed in different sections. Besides that, it might, while planning, have a patient diagnosed as an emergency patient. Generally, emergency patients are visited before regular patients in this model and have higher priority (Fairness). After doing some actions like visiting a doctor, the diagnosis may be based on the hospitalization of the patient, in which case the patient is referred to a hospitalized ward with a limited number of beds.

1 $${min}\,{\overline{c} }_{w}=\frac{1}{\sum_{i=1}^{n}{\tilde{w }}_{i}}\sum_{i=1}^{n}\left({\tilde{w }}_{i}.{CN}_{i}\right),$$

2 $${min}\,Z=\rho \sum_{i=1}^{n}\left({CN}_{i}-{r}_{i}\right),$$

3 $$\sum_{m=1}^{M}\sum_{l=2}^{L}{x}_{ijlm}=1, j=1,\ldots ,{h}_{i}, i=1,\ldots ,n,$$

4 $$\sum_{i=1}^{n}\sum_{j=1}^{{h}_{i}}{x}_{ijlm}\le {M}_{ijm} , l=2,\ldots ,L, m=1,\ldots ,M,$$

5 $${x}_{0j1m}=1, j,m=1,\ldots ,M,$$

6 $${C}_{1m}=\sum_{i}^{n}\left({x}_{i11m}.{r}_{i}\right)+\sum_{i}^{n}\left({x}_{i11m}.{\tilde{p }}_{i1m}\right), m=1,\ldots ,M,$$

7 $${t}_{1m}=\sum_{i}^{n}({x}_{i11m}.{r}_{i}), m=1,\ldots ,M,$$

8 $$\sum_{i=1}^{n}\sum_{j=1}^{{h}_{i}}{x}_{ijlm}\le \sum_{i=1}^{n}\sum_{j=1}^{{h}_{i}}{x}_{ij\left(l-1\right)m} ,m=1,\ldots ,M, l=3,\ldots ,L,$$

9 $${t}_{lm}\ge \sum_{i}^{n}\sum_{j=1}^{{h}_{i}}{(x}_{ijlm}.{r}_{i}), m=1,\ldots ,M, l= 2,\ldots ,L,$$

10 $${t}_{lm}\ge {C}_{\left(l-1\right)m} ,m=1,\ldots ,M, l=2,\ldots .,L,$$

11 $${t}_{lm}\ge min(\sum_{i=1}^{n}(\sum_{j=2}^{{h}_{i}}{x}_{ijlm}.\sum_{{m}^{^{\prime}}=1}^{M}\sum_{{l}^{^{\prime}}=2}^{L}({x}_{i\left(j-1\right){l}^{^{\prime}}{m}^{^{\prime}}}.\left({t}_{{l}^{^{\prime}}{m}^{^{\prime}}}+{\tilde{p }}_{i\left(j-1\right){m}^{^{\prime}}}\right)+\sum_{{i}^{^{\prime}}=0}^{n}\sum_{{j}^{^{\prime}}=1}^{{h}_{{i}^{^{\prime}}}}{x}_{i\left(j-1\right){l}^{^{\prime}}{m}^{^{\prime}}}.{x}_{{i}^{^{\prime}}{j}^{^{\prime}}\left({l}^{^{\prime}}-1\right){m}^{^{\prime}}} )))) , m=1,\ldots ,M, l=2,\ldots ,L,$$

12 $${C}_{lm}={t}_{lm}+\sum_{i=1}^{n}(\sum_{j=2}^{{h}_{i}}{x}_{ijlm}.{\tilde{p }}_{ijm}+\sum_{{i}^{^{\prime}}=0}^{n}(\sum_{{j}^{^{\prime}}=1}^{{h}_{i}}{x}_{i\left(j-1\right)lm}.{x}_{{i}^{^{\prime}}{j}^{^{\prime}}\left({l}^{^{\prime}}-1\right)m}))+{x}_{i1lm}.{\tilde{p }}_{i1m}+\sum_{{i}^{^{\prime}}=0}^{n}(\sum_{{j}^{^{\prime}}=1}^{{h}_{i}}{x}_{i1lm}.{x}_{{i}^{^{\prime}}{j}^{^{\prime}}\left({l}^{^{\prime}}-1\right)m}), m=1,\ldots ,M, l=2,\ldots ,L,$$

13 $$\sum_{m=1}^{M}\sum_{l=2}^{L}({x}_{i\left(j-1\right)lm}.{c}_{lm})\le \sum_{{m}^{^{\prime}}=1}^{M}\sum_{{l}^{^{\prime}}=2}^{L}{x}_{ij{l}^{^{\prime}}{m}^{^{\prime}}}.{t}_{{l}^{^{\prime}}{m}^{^{\prime}}}, j=2,\ldots .,{h}_{i}, i=1,\ldots .,n,$$

14 $$\left({x}_{ijlm}.{t}_{lm}.{s}_{i}\right)\le {x}_{{i}^{^{\prime}}{j}^{^{\prime}}lm}.{t}_{lm}.\left(1-{s}_{i}\right), j,{j}^{^{\prime}}=2,\ldots .,{h}_{i},\forall i,{i}^{^{\prime}},l=2,\ldots ,L, \forall m,$$

15 $$\left({x}_{ijlm}.{t}_{lm}.{y}_{ijlm}\right)\le {x}_{{i}^{^{\prime}}{j}^{^{\prime}}lm}.{t}_{lm}.\left(1-{y}_{ijlm}\right), j,{j}^{^{\prime}}=2,\ldots .,{h}_{i},\forall i,{i}^{^{\prime}},m=2,\ldots ,M, \forall l,$$

16 $$\sum_{l=2}^{L}{x}_{ijlm}\le {a}_{ijm},$$

17 $${CN}_{i}=\sum_{m=1}^{M}\sum_{l=2}^{L}{x}_{i{h}_{i}lm}.{c}_{lm}, i=1,\ldots .,n,$$

18 $$\sum_{i=1}^{n}\sum_{j=1}^{{h}_{i}}\sum_{m=1}^{M}\sum_{l=1}^{L}{z}_{ijlm}.{B}_{m}\le Bed,$$

19 $${x}_{ijlm},{z}_{ijlm},{y}_{ijlm}\in \left\{\text{0,1}\right\}, j=1,\ldots ,{h}_{i},i=1,\ldots .,n,m=1,\ldots ,M,l=1,\ldots ,L.$$

As mentioned above, Eq. (1) is one of the goal functions: to minimize patients’ mean total weighting time. Equation (2) is another purpose that reduces patient dissatisfaction due to increased patient waiting time in the hospital. Constraint (3) states that each patient’s operation can only be examined or processed in a single position in one ward. Constraint (4) ensures that the maximum number of people (operators) can be allocated to patient operations in each segment. Constraint (5) assigns zero position to each imaginary patient. Constraints (6) and (7) set the time of begin and finish of processing of the first position of each segment assigned to the imaginary patient, respectively. It equals zero. Also, these Constrains indicate that all units are available at zero moments and are ready to receive patients. Constraint (8) prevents a patient from assigning an operation to the position of a segment if the other area is empty. It constrains guarantees that patients will be sequenced in a single sequence. Constraint (9) indicates that the start time of each sequential position of the ward is more extended than when the patient was in the hospital. Constrain (10) suggests that each sequence’s start time is longer than the required for completing the place before that segment. Constraint (11) ensures that the start time of each sequence position is longer than the minimum time required to complete the primary operations of the patients being processed at that position. Since more than one operator per processor per section, the “minimum completion time” is set to visit the next patient as soon as the first operator is idle. Constraint (12) states that the time elapsed for each processing position in the ward is equal to when it began, plus the processing time for patients assigned to that position. Constraint (13) guarantees the processing of operations based on their sequence in each patient’s schedule, which is specified because of referral (or illness). The constrain means that the following process will not start until the prerequisite operation is completed and completed. Constraint (14) indicates the priority of emergency patients over other patients. This group includes patients who are initially admitted as emergency patients. This constraint guarantees that in all departments and situations, these patients will be visited before regular patients. Constraint (15) indicates the priority of emergency patients over other patients. This group includes patients who have been identified as emergency patients during the planning process. This constraint guarantees that in all departments and situations, these patients will be visited before regular patients. Limit (16) is applied to select the part to operate the pieces capable of showing it. Constraint (17) is considered to calculate the completion of patients’ operations according to the selected sequences. Constraint (18) ensures that the number of hospitalized patients does not exceed the available beds. Constraint (19) specifies the range of values allowed for the decision variable.

### Improving the procedure

In the proposed structure of the WOA algorithm, a recovery procedure is designed that applies and improves the solutions selected in the previous section. Improvement outputs are selected as the population of the next iteration of the algorithm. Implementation of the improvement procedure in this research is based on a variable neighborhood search (VNS). The VNS structure uses four Neighborhood Search Structures (NSS). These structures are used in VNS format, and their general structure is as follows Kardani-Moghaddam et al.^27^.
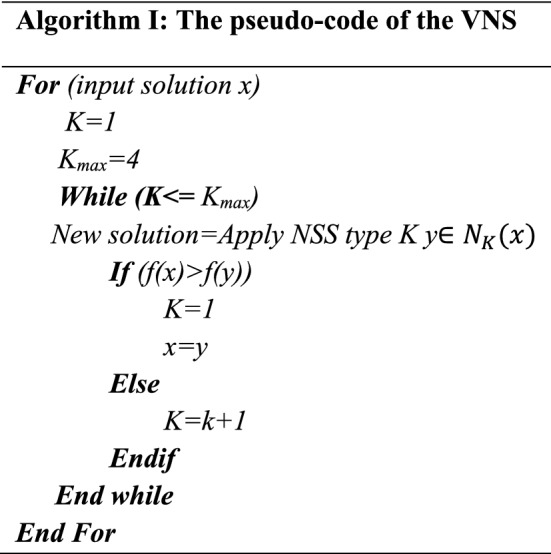


Each of the solutions in the population is answered to the VNS algorithm, and a response will be output. The corrective procedure will then be applied to the rest of the answer matrices. It will modify and replace the input answer as appropriate. The overall structure of the recovery procedure will be as follows:
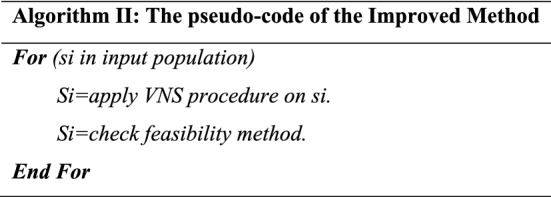


### Fairness algorithms in appointment scheduling

Fairness is an important issue to examine in appointment scheduling methods to give excellent service to patients arriving at various periods. In this part, the mathematical modeling is developed to include fairness. This section sets an NSGA-II (Non-dominated Sorted Genetic Algorithm) for handling sequential appointment scheduling with care delivery and fairness constraint (FCFC). NSGA-II is a well-known multi-objective optimization algorithm, quick sorting and powerful. Input parameters, including cutting speed, feed rate, flow rate, etc., are necessary conditions for optimizing the machining operations to reduce or maximize the machining efficiency.

#### Mutation operator

In this research, to implement the mutation operator, this paper used four neighborhood search structures described in the initial solution production section, such that a random number is generated between 1 and 4 and then given the production number One of the first, second, third, or fourth structures applies to the answer.

#### Intersection operator

The intersection operator designed in this algorithm is described below. This paper first assigns a number according to the patient number. For example, suppose this paper has four patients, each involving four operations. The number of wards in the hospital equals four. The procedure for assigning the number to the patients is shown below. In the following matrix, the numbers given below the operation are assigned an operation number. In this way, they are displaying the answers to combine the answers changes to three lines. Below is an instance of how to get creative with this line.

The differential between the maximum and minimum average waiting time, developed and implemented, is applied as a fairness constraint where α represents the acceptable threshold level.

20 $$\text{max}E[{W}_{i}^{n}]-\text{min}E\left[{W}_{i}^{n}\right]\le \alpha i\in \left[1,\ldots T\right].$$

The practical procedures of a scheduling policy for appointment scheduling with patient choice and service fairness in opinion as below:

Patients who refuse to admit the approval will be sent away. The method concludes each given call-in slot when no further profit is achieved by scheduling additional patients, or no intervals can be scheduled that satisfy the fairness requirement at the same time.

## Results

### Computational analysis

As mentioned, the present paper deals with the presentation and solution of the appointment scheduling problem model in the hospital. In this regard, a two-objective mathematical model concerning fairness in fuzzy conditions is presented. The WOA algorithm based on the Pareto Archive and NSGA-II algorithm has been used to solve the model in this research. In this part of the research, the results of model solving for various problems are presented. The performance of WOA and NSGA-II is benchmarked. To test the efficiency of the proposed whale algorithm, the algorithm was implemented in the MATLAB software environment^28^. The results of its implementation in a sample problem as a case study and practical problems done by GAMS version 24.9.1 software^29^ was compared with the results obtained from the NSGA-II algorithm. The remainder of this chapter describes the computational results. All the calculations were done with the i7 7500U 12 GB–1 TB–R5 M335 4 GB Core computer. Whale population size parameters, number of variable neighborhood search iterations and number of iterations in the whale optimization algorithm and population size parameters, mutation rate, intersection rate, and number of iterations in the NSGA-II algorithm are among these parameters. To adjust the settings of the whale algorithm, the values of each of these parameters are studied at three levels, which are shown in Table 1.

**Table 1:** Whale algorithm values in three levels.

Neighbourhood Search iterations	Population size	Number of the algorithm iterations
5	70	100
10	150	300
15	200	500

In addition, to adjust the parameters of NSGA-II, two settings of mutation rate and intersection rate at three levels and population size at three levels have been studied. These levels are shown in the following Table 2.

**Table 2:** Levels of NSGA-II parameters.

Population size	Intersection rate	Mutation rate	Iterations
70	0.75	0.006	150
150	0.85	0.009	300
200	0.95	0.01	500

Moreover, each problem is executed for each of the combinations mentioned above, and the gap criterion for each problem is computed. Finally, the corresponding graph is plotted. Also, for designing the parameter, the Taguchi experiment with the L9 method was used. The orthogonal table for the two algorithms is shown in Table 3.

**Table 3:** Orthogonal table for adjusting WOA’s parameters.

Test no.	Neighbourhood search repeat count	Population size	Iterations of the algorithm	RPD value
1	5	70	150	0.2341
2	5	150	300	0.4367
3	5	200	500	0.3395
4	10	70	300	0.3083
5	10	150	500	0.1285
6	10	200	150	0.2216
7	15	70	500	0.1993
8	15	150	300	0.4643
9	15	200	150	0.2942

**Figure 6:**
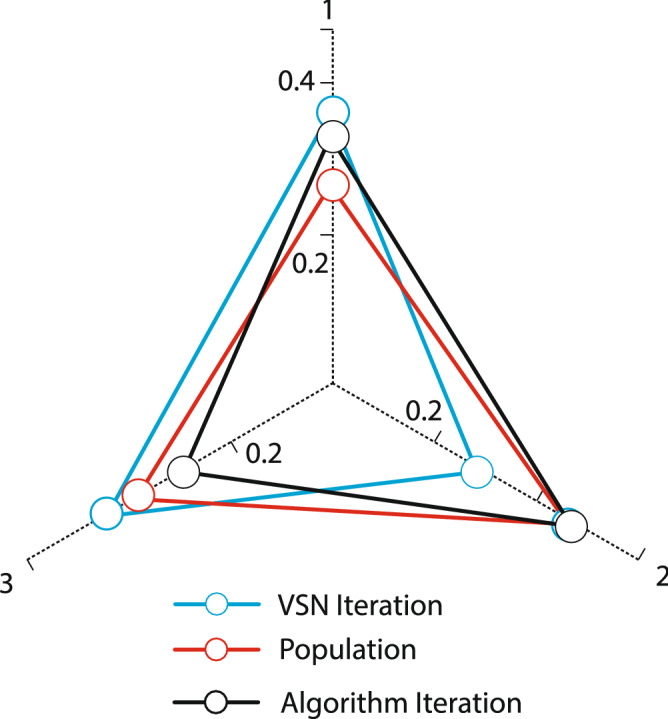
Average effect on WOA.

**Figure 7:**
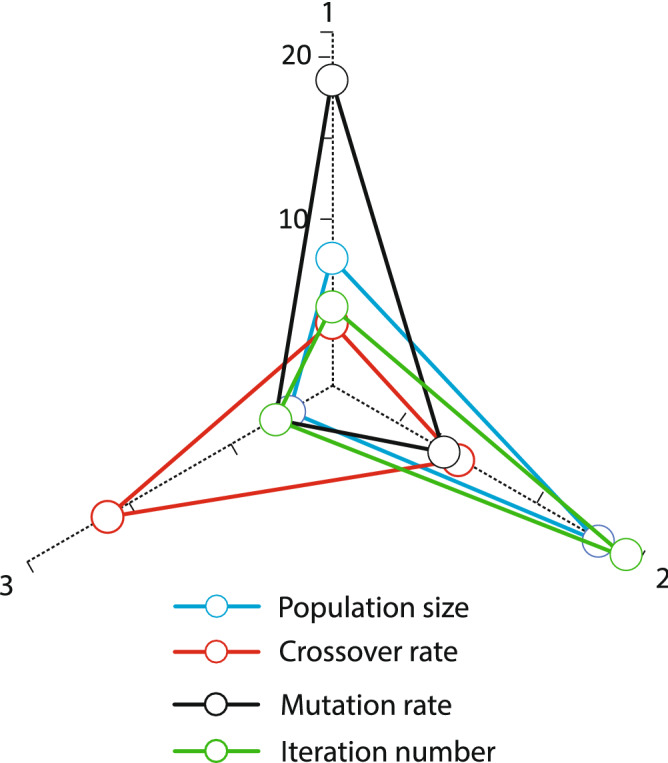
Noise signal in NSGA algorithm.

**Figure 8:**
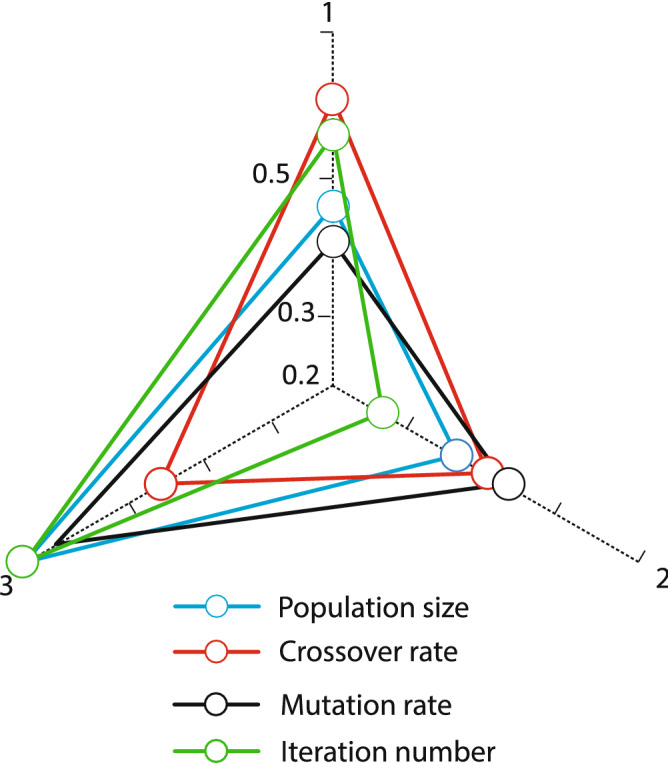
The average effect in NSGA algorithm.

Figures 5 and 6 illustrate the parameter analysis of the Taguchi method, as shown in Fig. 5. Also, where population size is at level 2, the number of algorithm iterations at level 2, and search Neighborhood is sufficient at level 1. In WOA, the population size is 150, the number of VNS repeats is 5, and the number of iterations is 300. Figures 7 and 8 show the Taguchi method for parameter adjustment, as seen in Fig. 7, level 3 for the jump rate and level 1 for the intersection rate; Level 3 is more effective for replicating the algorithm and population size. Therefore, 300 for population size, 500 for algorithm replication, 0.01 for mutation rate, and 0.75 for intersection rate are considered (see Table 4).

**Table 4:** Orthogonal table for setting parameters of NSGA-II algorithm.

Test no.	Population size	Intersection rate	Mutation rate	Iterations of the algorithm	RPD value
1	70	0.75	0.006	150	0.5032
2	70	0.85	0.009	300	0.1259
3	70	0.95	0.01	500	0.7419
4	150	0.75	0.009	500	0.6635
5	150	0.85	0.01	150	0.4917
6	150	0.95	0.006	300	0.0045
7	200	0.75	0.01	300	0.7124
8	200	0.85	0.006	500	0.7280
9	200	0.95	0.009	150	0.6521

### Numerical analysis

The objective of numerical methods is to examine the algorithm’s ability and provide some perspectives into facilitating realistic appointment scheduling. Firstly, two examples are given in this section to demonstrate the application of the two myopic scheduling algorithms. This paragraph analyzes the execution of the presented integrated whale algorithm and the NSGA-II algorithm to solve the designed problems. To evaluate the validity of the model, two small-scale issues are addressed by the whale algorithm. Details of the parameters and results of solving these two problems are presented below. The first problem consists of three patients and three sections, the parameters of which are shown in Table 5.

**Table 5:** Parameters of the problem three patients and three sections.

Patient	Operation	Processing time			${a}_{ijm}$		
		Section 1	Section 2	Section 3	Section 1	Section 2	Section 3
1	1	10	–	–	1	0	0
	2	–	9	–	0	1	0
2	1	–	6	–	0	1	0
	2	–	–	3	0	0	1
3	1	7	–	–	1	0	0
	2	–	–	4	0	0	1

It is assumed that the time of the patient’s arrival occurred at time 0. Of the three patients, patient 3 entered the emergency patient’s entrance. That is, patient 3 has a higher priority (Fairness).

It is also assumed that there is only one person in each department to serve and provide services.

Patients’ weight or preference are considered 2, 3, and 5, respectively.

The rate of decrease in patient satisfaction due to increased hospital stay is set at 0.2.

**Figure 9:**
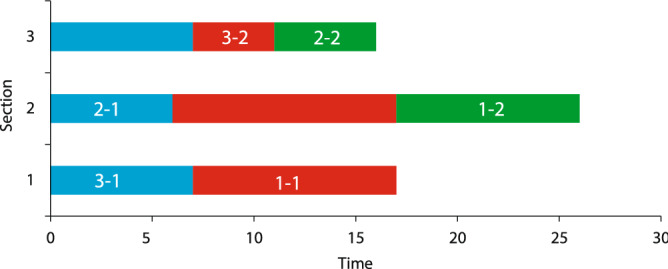
Optimal patient scheduling for the three patient and three ward problem.

As seen in Fig. 9, Patient 3, as an emergency patient, is admitted to the first and third ward before other patients. Completion times for patients 1 to 3 are 26, 14, and 11, respectively (see Table 6).

**Table 6:** Problem solving results in three patients and three sections.

Case	Status	${f}_{1}$	${f}_{2}$	
Schedule	${1}_{1}\to {3}_{1}, {1}_{2}\to {2}_{1}, {2}_{1}\to 0, {2}_{2}\to {3}_{2}, {3}_{1}\to 0, {3}_{2}\to 0$	15.5	25.5	$\text{min}{f}_{1},{f}_{2}$

Besides the mentioned problem, there is a further problem with 5 patients, and 4 units have been solved. The parameters related to this problem are presented in Table 7.

**Table 7:** Problem parameters 5 patients and 4 units.

Patient	Operation	Processing time				${a}_{ijm}$				${r}_{j}$	${w}_{j}$
		Section 1	Section 2	Section 3	Section 4	Section 1	Section 2	Section 3	Section 4		
1	1	–	97	–	–	0	1	0	0	0	3
	2	67	–	–	–	1	0	0	0		
	3	–	–	5	–	0	0	1	0		
2	1	86	–	–	–	1	0	0	0	0	3
	2	–	94	–	–	0	1	0	0		
	3	–	–	69	–	0	0	1	0		
3	1	–	77	–	–	0	1	0	0	0	4
	2	–	–	–	75	0	0	0	1		
	3	40	–	–	–	1	0	0	0		
	4	–	–	67	–	0	0	1	0		
4	1	–	18	–	–	0	1	0	0	0	2
	2	72	–	–	–	1	0	0	0		
5	1	–	4	–	–	0	1	0	0	0	1
	2	29	–	–	–	1	0	0	0		

It is assumed that the time of the patient’s presence occurred at time 0, and that of the three patients, patient 3 entered the door of emergency patients. That is, patient 3 has a higher priority with fairness.

It is also assumed that there is only one person in each department to serve and provide services.

The rate of decrease in patient satisfaction due to increased hospital stay is set at 0.2.

**Figure 10:**
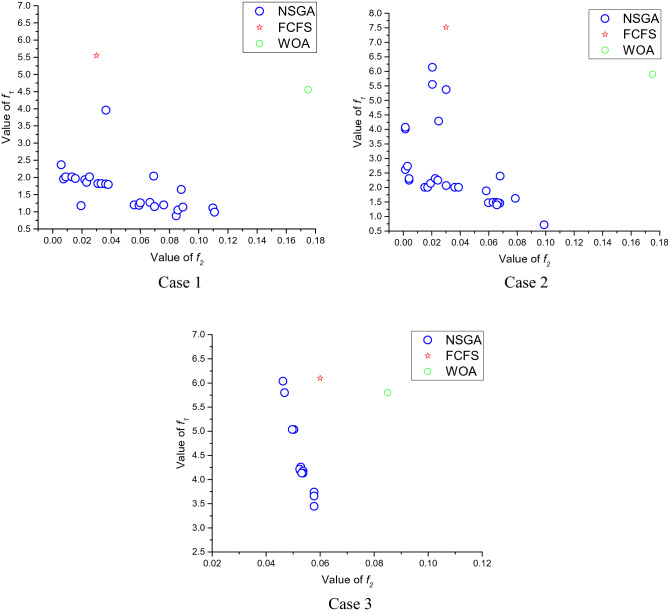
Distribution of objective function attributes in different theory situations.

From Table 8, NSGA-optimized approaches outperform FCFS and the WOA f1 approach in all five instances. The proposed NSGA meets the WOA Strategy on the second level but does not surpass the FCFS Strategy. Nevertheless, for its poor performance in f1, the FCFS approach is not successful. The results are consistent with the suggestion that the FCFS approach provides a fair system but does not consider capital. The implications of the greedy technique on f1 are because patients are admitted only based on the estimated duration of stage 2 but without any regard to the difference between the entries of various patient groups. Case 3 comes from a hospital, and the results in contrast with example 1 and case 2 are relatively odd. Therefore, an optimal strategy is difficult to achieve in this situation. In case 3, NSGA manages to outdo the WOA and the FCFS approach. However, their dominance is not as evident as in example 1 and case 2. The distribution of f1 and f2 values, in example 1 to case 3, is shown in Fig. 10. The target values of the strategies developed by NSGA, the FCFS strategy, and the WOA strategy are illustrated.

**Table 8:** Pareto answers from 5 patients and 4 sections.

Solution number	${f}_{1}$	${f}_{2}$
1	331.5	4269.5
2	333.5	4502.5
3	333.8	4339.5
4	334.3	3443
5	337.9	1810
6	342.2	2982
7	341.1	2283.5
8	343.7	2281.5
9	344.1	2190
10	345.3	1962
11	358.5	1910.5
12	360.4	1539

Figure 10 reveals that almost half of the solutions found by NSGA are better f1 and have better f2 than FCFS and WOA. Practically, all of the answers given by NSGA are better f1. Based on Fig. 10, considering the above, this paper suggests an NSGA designed to improve healthcare admission approaches for patients. This paper develops a chromosome coding scheme to optimize admission strategies to identify the assessment functions for two goals: efficiency and fairness. The introduced algorithms use hospital historical information. This paper, the algorithm proposed, will solve other difficulties with changed constraints without adjusting the coding strategy and the algorithm structure. Studies are carried out on three occasions, and the solution implemented by the proposed WOA is contrasted with the policy of justice and the strategy of NSGA. Numerical findings indicate that both the Fairness and WOA approaches are outperformed by the method optimized by the proposed algorithm. Most patients would be handled equally due to the fairness policy.

## Discussion

This study randomly generated two algorithms and solved several practical problems to compare two WOA and NSGA-II. Comparative results for solving these problems according to the indices presented in Tables 6 and 7 are shown. The following table shows the difficulties in n/m, where n represents the number of patients and m the number of wards. Also, for solving the sample problems, the model parameters are set as follows: In the fuzzy settings, to produce triangular numbers $({m}_{1}, {m}_{2}, {m}_{3})$, the first m2 is constructed, and then a random number r is generated in the interval (0,1) and m1 using the relationship of ${m}_{2}\times (r-1)$ and m3 with Using the relation ${m}_{2}\times (r + 1)$ will be produced. For determining fuzzy parameters, ${m}_{2}$ are randomly assigned, and two values of m1 and m3 are determined using MATLAB R2015a (MATLAB 8.5). For this reason, only the amount of m2 is set in the parameter setting of this parameter.

- Uniform distribution over the interval produces patients’ weights and weights of completion time [1, 9].
- Processing time for patient operations inwards is generated using a uniform distribution over the interval [100,1].
- The job preparation time is produced uniformly between 20 and 40% of the average completion time.

The comparative results in Tables 9 and 10 and the charts on the relative indices show that the whale algorithm is, in all cases, more capable of producing better quality responses than the NSGA-II algorithm. The whale algorithm can produce higher dispersion responses than the NSGA-II algorithm. In other words, the whale algorithm is more capable of detecting and extracting the active region than the NSGA-II algorithm. As shown in the tables above, the NSGA-II algorithm produces higher-order solutions than the WOA. The epsilon constraint method produces Pareto optimal solutions to variable and linear scheduling problems in which all criteria and decision variables take on numerical values. The suggested method is illustrated with four benchmark functions in addition to the WOA and NSGA methodologies in the health appointment scheduling problem. The epsilon constraint method and its variants have been applied in many systems and applications described as an essential way to solve multi-objective linear programming problems and preferred over competing techniques. However, it comes down in this research in the camper to WOA. These weaknesses are summed up by the useless managing of the actual quality and Pareto analysis statements of the optimal solutions and the significant time required to implement it as more objective functions are introduced to a problem. The epsilon constraint algorithm optimizes one objective function while treating all other objectives as constraints. Although the model is widely used for multi-objective linear programming problems based on computational and results, we still limit ourselves to using WOA to solve this constraint as more benefits are obtained.

**Table 9:** Results for solving small size issues.

Prob.	WOA (whale’s optimization algorithm)					NSGA-II					ε-constraint method				
	Quality	Spacing	Diversity	CPU time	Pareto Ans	Quality	Spacing	Diversity	CPU time	Pareto Ans	Quality	Spacing	Diversity	CPU time	Pareto Ans
3/4	85.2	0.92	985.2	155.2	380	14.8	0.78	740.7	73.4	301	27.2	0.88	832.4	81.2	301
5/4	83.5	0.51	1365.9	159.2	299	16.5	0.47	840.9	73.6	79	22.5	0.67	847.1	86.6	87
7/4	88.1	0.64	1439.9	160.1	534	11.9	0.56	850.2	80.1	47	16.2	0.60	870.2	90.1	64
10/4	100	1.06	1468.3	162.5	200	0	0.71	1130.6	89.2	301	0	0.91	1234.1	95.5	280
12/4	87.7	0.68	1582.2	163.1	198	12.3	0.44	1220.4	85.2	217	9.3	0.54	1256.2	97.8	225
16/4	87.6	0.91	1702.3	171.8	231	12.4	0.78	1261.3	105.7	211	6.3	0.88	1265.8	103.7	321
18/4	83.4	0.71	1708.9	181.8	187	16.6	0.47	1349.1	112.6	149	4.8	0.54	1367.7	109.4	102
18/5	85.8	0.73	1763.2	182.4	345	14.2	0.62	1360.6	124.5	348	12.1	0.65	1370.3	114.9	349
18/7	88.1	1.01	1930.2	184.7	488	11.9	0.49	1281.4	124.9	351	10.5	0.89	1381.6	123.6	380
20/7	88.7	1.32	2012.9	199.4	529	11.3	0.70	1495.4	136.7	400	7.2	0.98	1470.4	130.1	413

**Table 10:** Results for solving problems of large and medium-size.

Prob.	WOA (whale’s optimization algorithm)					NSGA-II					ε-constraint method				
	Quality	Spacing	Diversity	CPU time	Pareto Ans	Quality	Spacing	Diversity	CPU time	Pareto Ans	Quality	Spacing	Diversity	CPU time	Pareto Ans
25/5	90	0.75	2871.6	424.4	299	10	0.74	1901.6	179.2	161	47.3	0.82	1882.1	180.2	122
30/5	85.9	1.72	2685.3	427.8	321	14.1	0.64	1954.2	235.9	208	67.8	0.34	1837.8	185.6	183
40/5	87.6	1.67	3063.5	440.3	407	12.4	0.76	2112.5	354.4	198	64.2	0.80	1890.9	198.1	264
50/5	70.9	0.73	2636.3	459.2	513	29.1	0.65	1901.9	386.5	192	46.7	0.11	2234.1	199.5	240
60/5	89.9	0.71	2816.5	568.8	376	10.1	0.70	2265.1	397.7	211	79.2	0.18	2257.3	245.8	223
70/5	66.8	1.70	3486.3	601.8	322	33.2	0.54	2796.6	429.4	319	55.3	0.88	2269.1	309.7	311
80/5	87.2	1.17	4121.9	614.1	285	12.8	0.65	3278.6	437.9	200	64.4	1.24	3377.7	336.4	205
90/5	100	1.13	4565.9	769.2	309	0	0.64	3397.7	543.4	188	74.8	1.05	3390.4	418.9	329
100/5	88.4	1.04	5054.1	783.6	300	11.6	0.73	4758.7	650.2	197	46.2	0.99	4345.6	527.6	290
150/5	85.1	1.75	6077.6	808.7	398	14.9	0.56	5779.7	750.6	320	70	1.28	5438.3	689.1	218

As seen in the following Tables 9, 10 and 11, the runtime of the algorithms show that runtime values and runtime comparisons indicate that the multi-objective WOA has a higher resolution time. Because of the proposed structure of the proposed method, this method intelligently searches many points of the answer space in each iteration. This method consumes more computational time than the NSGA-II method. According to the comparison findings, the WOA can supply higher-quality replies than the NSGA-II algorithm. The whale method is better capable of identifying and retrieving the active region than the NSGA-II algorithm. It can provide more excellent dispersion responses than the NSGA-II algorithm. Also, Low divergence and simple localization are downsides of the WOA compared to the NSGA. However, the WOA’s advantage is clear. The neglect demonstrates that the WOA algorithm used in the fairness application outperforms the regular NSGA in both global and local searches.

**Table 11:** The comparison between the models.

Authors	Model	Solution	Optimal function	Results
Rashid et al. (2020)	Hybridized WOAGWO with Solution Approach	Solving critical probability in pressure vessel design in hospital	Statistical test compute for unimodal and multimodal functions	Shows that WOAGWO outperforms other algorithms depending on the test
Oliva et al. (2020)	Mixed integer linear programming (MILP), two binary variants of WOA	Minimized cost feature and total waiting time	A particle swarm optimizer and a mixed statistical test called	Achieved the smallest number of selected features with the best classification accuracy in a minimum time
Tahir et al. (2020)	Implementing two stages, binary chaotic (BCGA) and WOA	Two stages fitness function and BCGA feature given significant classification accuracy	Evolutionary computing-based optimized patient’s waiting time	BCGA map perform better and find a robust subset as compared to other maps in enhancing the performance of raw WOA
The presented method	Inter Linear Programming and WOA technique	Resolves appointment scheduling and waiting time problems for effective FCFS policy and obtained patients satisfaction	Numerical results indicate that both the FCFS and WOA approaches are strategy optimized	Both the FCFS and the WOA strategies data to gain the most propriety (Fairness) results and patient satisfaction

## Conclusion

Overall, the present paper dealt with the presentation and solution of the appointment scheduling problem model in the hospital under the fairness approach. A two-objective mathematical model concerning fairness in fuzzy conditions was presented. WOA and NSGA-II algorithms were employed to solve the model as two multi-objective optimization algorithms. In addition to these two algorithms, this paper discussed multiple strategies in appointment scheduling. This paper has calculated WOA and NSGA with different hypotheses to satisfy the evaluation and patient-related variables in hospitals and the final part of the model. Finally, this paper has three case studies on NSGA and WOA plus a fairness strategy. Then this paper computed the FCFS to find the most crucial element obtained from the figure to scenarios optimized by the proposed NSGA contrasted with the FCFS and WOA. Experimental results show that the approach optimized by the proposed algorithm outperforms both the FCFS and the WOA strategies.

Besides those algorithm optimizations for this paper, this paper discussed a simulation model for two types of patients in normal and emergency conditions. In contrast, it has been modeled in Any Logic simulation software based on doctors, beds, and other services. In the simulation section, this paper found that the fairness objectives for each patient in the settings option were set at zero for regular patients and 2 for emergency patients. In emergency patients, these two numbers are optional and can only be considered a higher amount. Since there are only two groups of patients, whatever amount is found will not make any difference. Then this paper simulates two types of patients with some random default data to gain the most propriety (Fairness) results and patient satisfaction. Knowing this final results efficiency comes out, this paper calls the system run with the values of the initial parameters run 0. At this stage, it is observed that this paper has about one efficiency in the acceptance section. To determine if an increase in the number of admissions can reduce waiting times and keep performance at a high level, this paper will increase the number of individuals in the admission department in a few steps to maximize the number of people who can keep getting high. Other uncertainties can be added to the models for future research, such as available time of service and patients in urgency. Several case studies on the subject of simulation optimization explain lognormal service time. To what extent it will affect the income, and the optimum allocation of intervals remains unclear. Another possible direction would be to model the correlations between various doctors. The free doctor will help diagnose some of the patients when one doctor is loaded. Cooperation also occurs when the data is released among doctors. Patients may pick a different doctor instead of the primary care provider appointed.

## Abbreviations

*N*: Numbers of patients
*i*: Patient index
*S*_*i*_: Patient condition mode 1 = Emergency/0 = normal
*M*: Number of departments (admission, triage, pharmacy, lab, etc.)
*j*: Number of operations related to patients
*B*_*m*_: If it is possible in *m* section to diagnose hospitalization = 1/otherwise = 0
*Be*: Number of beds in each section
*r*_*i*_: *i*Th patient arrival time
*O*_*ij*_: *j*Th operation of *i*th patient
*m*_*ijm*_: The number of people in the *m*th department who can process the patient for *o*_ij_ operations
$\tilde{p}_{ijm}$: Fuzzy processing time *O*_*ij*_ operation in section *m*th
$a_{ijm}$: This parameter indicates the permissibility of processing *O*_*ij*_ in the *m*th section, which equals 1 if the *O*_*ij*_ operation is permitted in the *m*th section; otherwise, it is 0.
*l*: Indicates the position or queue in sections
$\rho$: Decreased patient satisfaction rate due to increased overwriting and cost
$\tilde{w}_{i}$: The rate of *i*th patient fairness is fuzzy

## Decision variables

$x_{ijlm}$: The parameter is the same as 1 if the action *O*_*ij*_ is done in section *m*_th_ at position *i*th. Otherwise, it is equal to 0.
$y_{ijlm}$: The parameter is the same 1 if the action *O*_*ij*_ is done in section *m*_th_ at position *i*th emergency patient; otherwise, it is equal to 0.
$z_{ijlm}$: The parameter is the same 1 if the action *O*_*ij*_ is done in section *m*_th_ at position *i*th the diagnosis is based on the patient’s hospitalization; otherwise, it is equal to 0.
$t_{lm}$: The start time of the practical processing that is in the *l*th position of the *m*th part
$c_{lm}$: The end time of the practical processing that is in the *l*th position of the *m*th part
$t_{lm}$: The start time of the practical processing in the *l*th position and the *m*th part
